# Identification of Volatile Compounds in Blackcurrant Berries: Differences among Cultivars

**DOI:** 10.3390/molecules26206254

**Published:** 2021-10-15

**Authors:** Sandy Pagès-Hélary, Laurence Dujourdy, Nathalie Cayot

**Affiliations:** 1 AgroSup Dijon, Université Bourgogne Franche-Comté, PAM UMR A 02.102, F-21000 Dijon, France; sandy.pages-helary@agrosupdijon.fr; 2 Agrosup Dijon, Service d’Appui à la Recherche en Science des Données, F-21000 Dijon, France; laurence.dujourdy@agrosupdijon.fr

**Keywords:** blackcurrant berries, cultivars, volatile compounds, GC-MS, SPME, multivariate statistical analyses, chemical profiling

## Abstract

Berries of blackcurrant are known to produce a strong flavor. Some previous studies have reported that a given cultivar of blackcurrant can produce berries with a specific profile of volatile compounds. For the Burgundy region in France, the Noir de Bourgogne cultivar is especially important because it is the main ingredient of a liquor with a designation of origin. The aim of the present study was to characterize the volatile fractions of berries from 15 cultivars in order to explore the possibility of using different cultivars for liquor production. The plants were cultivated under the same conditions and harvested in the same year. The volatile fractions of the harvested berries were analyzed using HS-SPME-GC-MS. Thorough univariate statistical analysis and multivariate analysis were applied to the dataset, which made it possible to identify groups within cultivars. The Rosenthal cultivar exhibited a quite flat profile; the Lositkia, Ben Tiran, and Barchatnaia cultivars shared common features; the Noir de Bourgogne cultivar showed the highest amounts of molecules such as 3-carene, limonene, β-phellandrene, ocimene, α-terpinolene, and bicyclogermacrene. None of the studied varieties were close to the Noir de Bourgogne on the basis of VOC analysis.

## 1. Introduction

Blackcurrant berries (*Ribes nigrum* L.) are cultivated in different countries around the world for diverse uses; for instance, as perfumes, jams, or juices or as coloring matter or an ingredient for alcoholic beverages [1]. In France, blackcurrant is mainly cultivated in four different areas: Val de Loire, Oise, Vallée du Rhône, and Burgundy. For the Burgundy region, blackcurrant is especially important because it is the main ingredient of blackcurrant liquor, which is an emblem of this area. The liquors cassis de Dijon and cassis de Bourgogne both hold designations of origin. In order to get these designations of origin [2,3], liquors have to be produced mainly from the Noir de Bourgogne cultivar, which is responsible for the special taste of these liquors.

The particular organoleptic quality of the Noir de Bourgogne cultivar is empirically well-known but there are limited data in the literature to demonstrate this, except for the publication by Latrasse et al. [4].

In fact, Latrasse et al. [4] studied the volatiles of a blackcurrant juice distilled under vacuum and of hydro-alcoholic infusions. They reported that the Noir de Bourgogne cultivar was particularly rich in diacetyle, ethyl butyrate, and eucalyptol. No other data have been further reported for this cultivar. The more recent studies have been published on other cultivars used throughout the world; for example, the Brödtrop cultivar was used in the study by Liu et al. [5] and found to be characterized mainly by ethyl acetate, aromadendrene, *p*-cymen-8-ol, 3-methyl-1-butanol, and *cis*-3-hexen-1-ol. The cultivars Mortti, Ola, and Melahati were used in the study by Marsoll-Vall et al. [6], who reported monoterpene hydrocarbons and oxygenated monoterpenes as the major classes of volatiles.

The volatile fraction of blackcurrant berries is known as being sensitive to the cultivar, but it is also influenced by the climate, the area of growth, and processes like freezing or juice extraction [7]. Therefore, great attention should be paid to these parameters and to experimental conditions when comparing the volatile fractions of different cultivars.

The present study focused on the volatile fraction of blackcurrant berries from different cultivars, including Noir de Bourgogne. The aim was to characterize the volatile fractions and compare them to that of the Noir de Bourgogne cultivar in order to explore the possibility of using other cultivars for the liquor production. The substitution would be possible if a cultivar showed organoleptic qualities close to those of Noir de Bourgogne and better resistance to pests and climate changes.

The similarities and differences among the volatile fractions of the blackcurrant berries from fifteen different cultivars (including Noir de Bourgogne), cultivated under the same conditions (same area, same treatment, etc.) and harvested in the same year, were studied. Odorant molecules from all the different berries were collected and identified using HS-SPME coupled with GC-MS. The whole dataset was analyzed with different statistical methods in order to determine differences. The objective was to obtain chemical profiles for the volatile organic compounds (VOC) of the blackcurrant berries of the different cultivars.

## 2. Results and Discussion

### 2.1. Volatile Compounds Representative of the 15 Cultivars

Molecules detected and identified in the headspace for the berries from the 15 cultivars are listed in Table 1.

Only three compounds were badly chromatographed due to co-elution, but identification was reliably achieved by probability-based matching with mass spectra in an MS library:

- For retention index 1203, the peak was attributed to β-phellandrene;
- For retention index 1256, the peak was attributed to ocimene;
- For retention index 1622, the peak was attributed to aromadendrene.

Additionally, three compounds could not be identified and were named NI1, NI2, and NI3 (respectively, retention indexes 1162, 1587, and 1825).

Other than these volatiles, 31 compounds were clearly identified, among which 26 were already reported in the scientific literature. Thus, 34 compounds were retained, which are reported in Table 1.

The profiles for volatile compounds varied greatly among the different cultivars, and they could be used to associate a specific odor with the corresponding berries.

**Figure 1:**
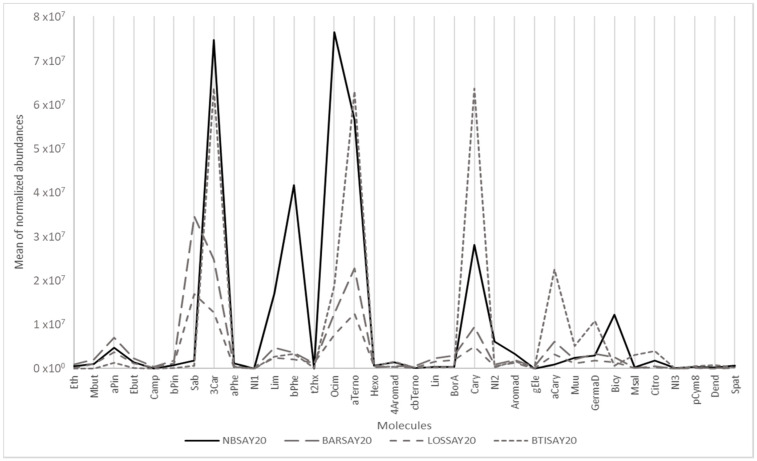
Graphical representation of the main odorant volatiles extracted from berries of different cultivars.

For example, Figure 1 shows the mean of the normalized abundance of the 34 targeted compounds for four cultivars showing very different profiles, among which is Noir de Bourgogne (NBSAY20). Limonene, β-phellandrene, ocimene and bicyclogermacrene were more abundant in Noir de Bourgogne than in the three other cultivars. Descriptors were taken from www.thegoodscentscompany.com and www.pherobase.com, (accessed on 17 September 2021). The odor descriptors of these compounds were respectively citrus, mint/herbal, floral, and green/woody/weedy. The chemical profile for the Ben Tiran (BTISAY20) cultivar had the highest abundances of α-terpinolene, caryophyllene, α-cayophyllene, germacrene D, and citronellol. The odor descriptors of these compounds were respectively woody/pine/citrus, woody/clove, woody, wood/spice, and floral/rose bud/citrus.

In contrast, the VOC profiles of the Barchatnaia (BARSAY20) and Lositkia (LOSSAY20) cultivars were quite flat except for sabinene, described as woody/citrus/pine/spice.

### 2.2. Data Sorting

From the chromatographic study, a dataset with 45 observations (15 cultivars × 3 replicates) and 34 variables (peak areas of detected volatile compounds) was obtained.

Prior to the establishment of a GC volatile fingerprint, the reproducibility of the peak areas was checked using the relative standard deviation (RSD). This was calculated from triplicates by multiplying the standard deviation by 100 and dividing by the mean value of the peak areas (given in Table 1).

Two types of RSD were calculated and named RSD “between” and RSD “within”.

The RSD “between” corresponded to the variation coefficient between different cultivars. It made it possible to determine whether there were huge or small differences between cultivars for each volatile compound.

The RSD “within” corresponded to the variation coefficient between replicates of a given sample (=cultivar) for each molecule. It made it possible to determine whether the peak area of a given molecule was repeatable or not. These calculations helped to target molecules that were “stable” when similar chemical profiles were analyzed.

The RSD provided information on the extent of variation of a compound. The rarity of compounds, calculated using the frequency of appearance (presence or absence in the samples) in a dataset, made it possible to evaluate discriminating power. Indeed, if a compound was present only in a particular cultivar then it was considered typical of this cultivar (Table 1).

The RSD “within” and RSD “between” are reported in Table 2 for each compound and each cultivar. The extent of variation in a peak area (characterizing its stability) was based on the following assumption. On one hand, the variability within the peak area was considered high if the RSD “within” was above 40%. This number was chosen because of the complex matrix and the difficulty of integrating the peak. It meant that the chromatography of the compound was not reproducible. With such an assumption, ethanol, methyl butanoate, and ethyl butanoate were eliminated from the list of target compounds.

On the other hand, if the RSD “between” was below 70% (chosen for the same reason as for the RSD “within”), this meant that the compound did not vary sufficiently between samples to be able to differentiate them by cultivar. With such a rule, camphene, α-phellandrene, NI1, hexanol, NI3, and *p*-cymen-8-ol were eliminated. In parallel, we checked that there were no outliers. The RSD “between” values were examined in conjunction with the frequency of the appearance of target compounds between different samples. If a peak was rarely present and its area had a high variability, the compound was considered as discriminant. For example, camphene had a low appearance frequency (33%), an RSD “within” far lower than the limit (18), and an RSD “between” value of 69. Therefore, it was kept in the list of target molecules. At the end of this stage, we decided to eliminate only five molecules: ethanol, methyl butanoate, ethyl butanoate, NI1, and hexanol.

Then, chemical profiles of cultivars were obtained with 29 target molecules and an exploratory statistical study was implemented. In order to further reduce this number of target compounds, an initial PCA was undertaken, and γ-elemene and NI3 could be eliminated. We also checked that these compounds had tangent RSD values.

Finally, descriptive analysis (univariate and multivariate) was implemented with the 27 remaining target compounds.

### 2.3. Differences among Cultivars Using Univariate Descriptive Analysis

**Figure 2:**
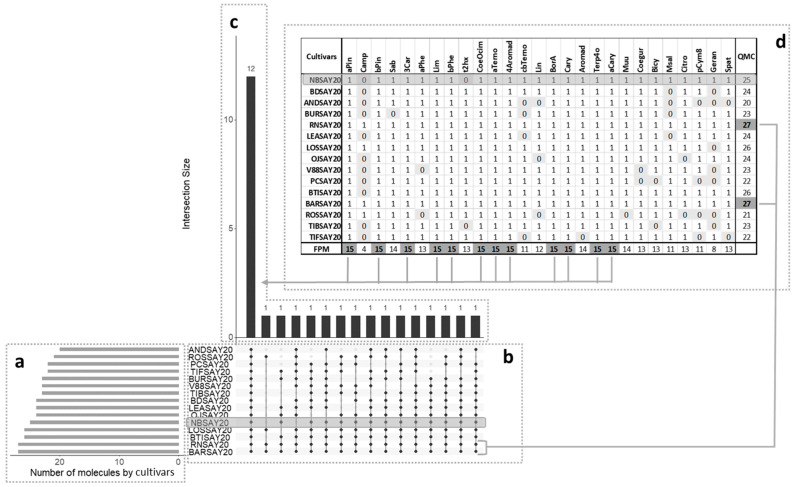
UpSet plot showing co-occurrence of volatile molecules in the various cultivars. QMC: Quantity of molecules found in each cultivar. The total size of each set is represented on the left barplot (**a**). Every possible intersection is represented by the bottom plot (**b**), and their occurrence is shown on the top barplot (**c**). (**d**) Detailed table with frequency of presence of molecules (FPM) in cultivars; presence is indicated by 1 and absence by 0.

Data visualization was used to find patterns within complex datasets with many features. An UpSet plot (Figure 2) [17] was used to group data points that had many of the same values across different features together and find the largest intersecting sets. The plot has several parts.

The central bar chart (Figure 2a) shows the number of compounds in the data that were held in common in the cultivars. Each bar indicates a different combination. Underneath the bar chart, a graphical table (Figure 2b) shows which cultivars the different combinations of compounds concerned. Each row indicates one of the fifteen cultivars. The black dots and lines show the combinations of cultivars that made up subsets of compounds. Reading from left to right, all cultivars had 12 compounds in common. The corresponding compounds are in the table above the bar chart (Figure 2c). The molecules showing a frequency of presence of molecule (FPM) of 15 in a cultivar were the ones present in all cultivars: α-pinene, β-pinene, 3-carene, limonene, β-phellandrene, ocimene, α-terpinene, 4-aromadendrene, bornyl acetate, caryophyllene, aromadendrene, and α-caryophyllene. The smaller bar chart (Figure 2d) shows the unconditional frequency count (quantity of molecules (QMC)) of each cultivar across all subsets. It can be observed that the highest value corresponded to the Royal de Naples and the Barchatnaia cultivars, which had all the target compounds, and the lowest value was that of the Andega cultivar with 20 compounds. The Noir de Bourgogne cultivar had 25 compounds, including 12 in common with the other cultivars. All the other compounds of the Noir de Bourgogne cultivar were also found in at least seven other cultivars in the present study. The Noir de Bourgogne cultivar presented a medium number of molecules compared to the other cultivars and was not characterized by specific molecules.

**Figure 3:**
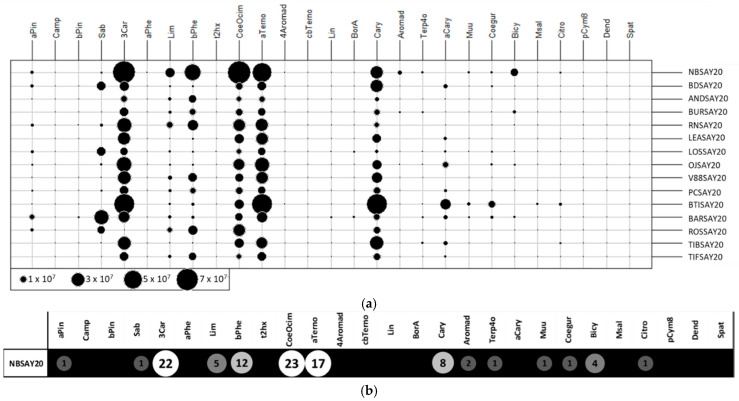
Two-dimensional table plot with proportional circles for peak areas of each volatile compound: (**a**) raw data and (**b**) relative abundance.

A two-dimensional table plot [18] with a proportional circles for the peak area of each compound is shown in Figure 3a (raw data). Missing values were replaced by the detection limit value, set to 50,000 (corresponding to 100,000 divided by 2 g of sample).

The cultivars Noir de Bourgogne and Ben Tiran presented compounds with larger circles than other cultivars. Some molecules were more intense for the Noir de Bourgogne cultivar: 3-carene, limonene, β-phellandrene, ocimene, NI2, and bicyclogermacrene. Figure 3b presents the chemical profile of the Noir de Bourgogne cultivar with relative abundances indicated by the circles. This figure underlines the importance of 3-carene, ocimene, and α-terpinolene in the fingerprint of the Noir de Bourgogne cultivar.

### 2.4. Differences among Cultivars Using Multivariate Descriptive Analysis

In order to explore in more depth the chemical profiles and determine the existence of specific groups, we first used principal components analysis (PCA) on normalized data (each peak area was divided by the sum of the areas of all peaks), as a flexible tool. The values for the total soluble solids (°Brix) of the blackcurrant berries, which were not obtained from the GC-MS analysis, were added as additional data (Appendix A) in order to visualize the relationship of this variable with the set of active variables via the factorial axes.

**Figure 4:**
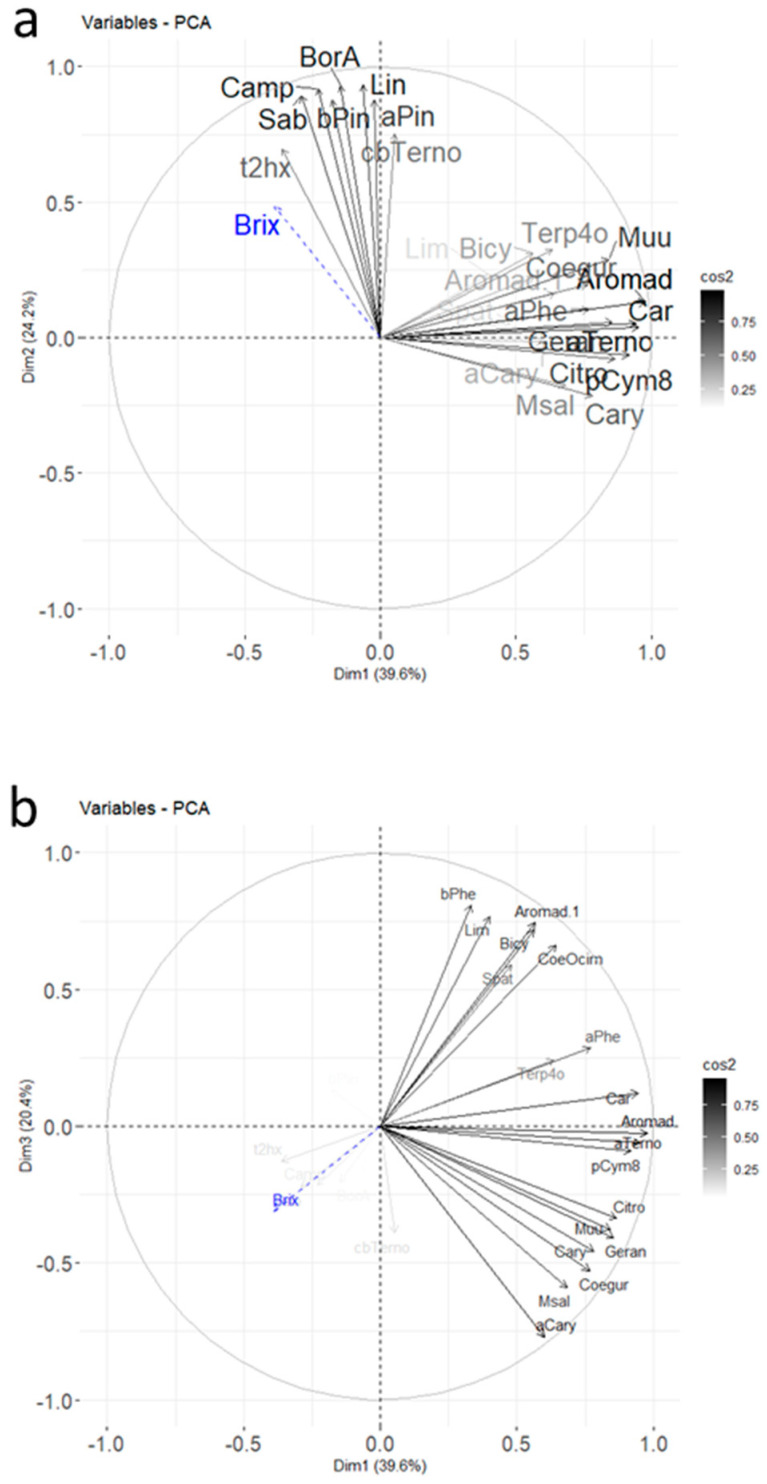

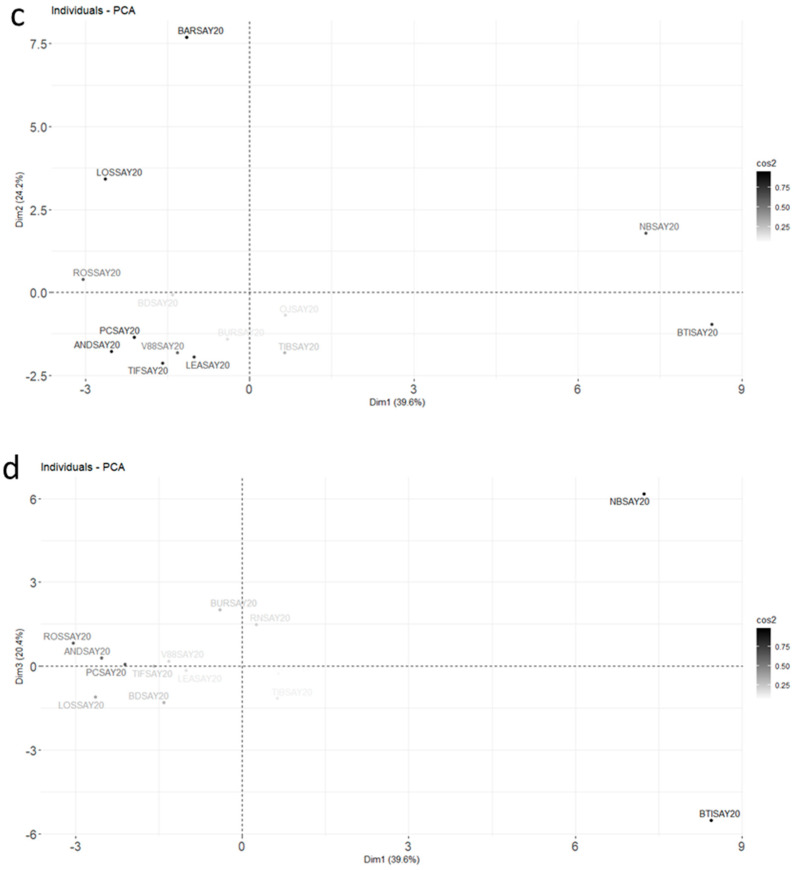
Final PCA plot released for harvest data from 2020 for the molecules retained after RSD sorting and the first PCA (Car indicates 3Car and Aromad indicates 4Aromad). The color gradient represents the level of square cosines for the variables (**a**,**b**) and the individuals (**c**,**d**). Variables with poor representation (cos^2^ < 0.5) are invisible. The supplementary variable, the Brix degree, is in dashed blue; (**a**,**b**) correspond to the variable plots in plans Dim 1–Dim 2 and Dim 1–Dim 3, respectively; (**c**,**d**) correspond to the individual plots in plans Dim 1–Dim 2 and Dim 1–Dim 3, respectively.

The PCA with correlations and a mapping of molecules and cultivars is shown in Figure 4.

Three dimensions accounted for 80.7% of the total variance. The contributions for the different molecules highlighted in Figure 3 for the Noir de Bourgogne cultivar were checked: limonene was well-represented in dimension 2, and 3-carene and α-terpinolene were well-represented in dimension 1. Ocimene, NI2, and bicyclogermacrene were well-represented in the plan Dim 1–Dim 3. The Brix degree had a poor representation; its impact was low compared to the blackcurrant composition in volatiles. It did not explain the differences observed between varieties of blackcurrant by itself.

Different groups of cultivars could be observed in the Dim 1–Dim 2 plan. The Barchatnaia, Lositkia, and Ben Tiran cultivars formed three independent groups. In the Dim 1–Dim 3 plan, the Noir de Bourgogne and Ben Tiran cultivars were opposed on axis 3. Noir de Bourgogne was characterized by high peak areas for the group of variables comprising β-phellandrene, limonene, aromadendrene, bicyclogermacrene, and ocimene, while Ben Tiran had high peak areas for α-caryophyllene, germacrene D, and methyl salicylate. Noir de Bourgogne had a unique profile and no other cultivar could be grouped with it.

**Figure A1:**
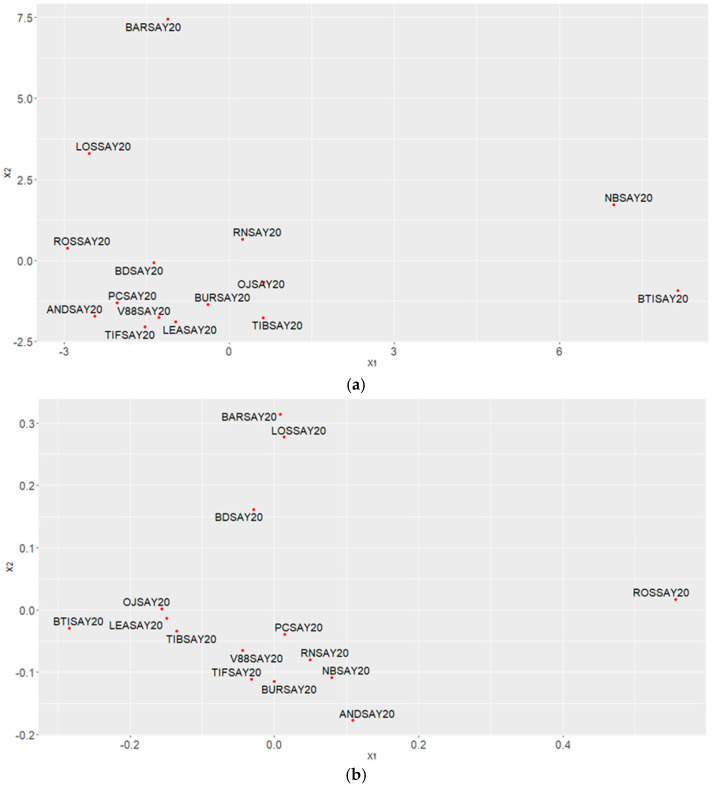
Multidimensional scaling using (**a**) Euclidean distance and (**b**) 1—Pearson correlation coefficient (1—cor).

MDS was carried out using Euclidean distances (see Appendix A, Figure A1a) and a distance associated with a Pearson correlation coefficient d = 1 − cor (see Appendix A, Figure A1b). From the joint analysis of both graphs, we concluded that the Noir de Bourgogne cultivar was opposed to the Lositkia cultivar, and the Rosenthal cultivar was opposed to the Ben Tiran cultivar. Finally, the Barchatnaia and the Lositkia cultivars shared a common feature: high peak areas for Camphene, Sabinene, Linalool, and Bornyl Acetate.

The Lositkia, Ben Tiran, and Barchatnaia cultivars showed quite rich profiles (26 or 27 molecules) and had 25 molecules in common. Some of them were intense (high peak areas) and the Ben Tiran cultivar exhibited the most intense profile.

## 3. Materials and Methods

### 3.1. Cultivars and Cultivation

**Figure 5:**
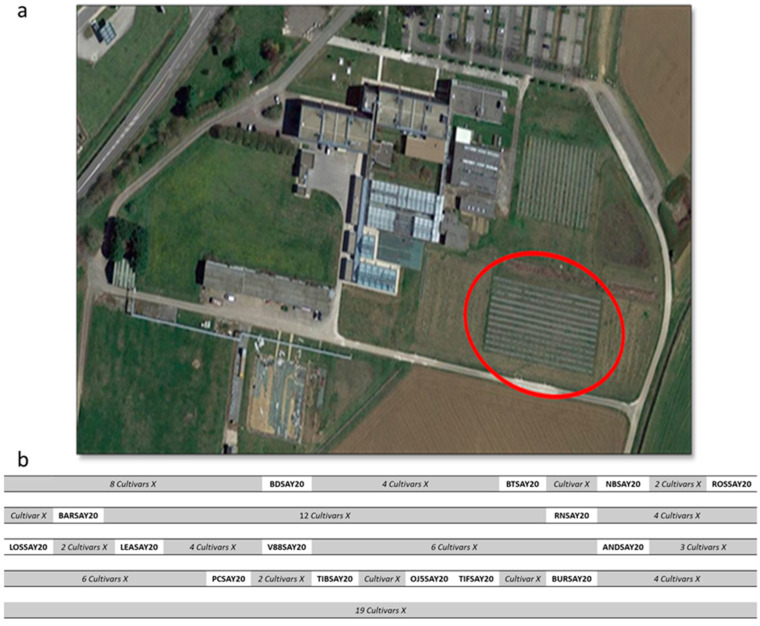
Cultivars and growing areas. (**a**) Aerial view of the Sayens site of Agronov, Agricultural Chamber of Burgundy, Bretenières, France. The red circle indicates the plot where the fifteen blackcurrant cultivars were grown; (**b**) the organization of the plot. “Cultivars X” refers to other cultivars from the Sayens collection that were not taken into account in the present study.

Fifteen cultivars of blackcurrant (Table 3) were grown in an open field on an experimental plot located in Burgundy (France, 47°14′6.508″ N, 5°6′28.096″ E), as shown in Figure 5a. For each cultivar, three plants were cultivated and the plot was organized as described in Figure 5b.

All plants were about six years old and were pruned each year in winter around January (only dead and obstructing branches were cut). Each blackcurrant plant received fertilizers (nitrogen, potassium, magnesium, and phosphorus) but not watering or chemical weeding.

### 3.2. Berry Harvesting and Storage

The fifteen cultivars of blackcurrant were harvested at maturity. Three conditions had to be met for the fruit to be considered ripe: the berries had to be easy to pick, they had to color the skin when crushed between two fingers, and the total soluble solids concentration (°Brix) at harvest had to be over 15°. As all the cultivars ripened at different times (early ripening, middle-season ripening, or late ripening), the harvesting period extended over one month from June to the beginning of July, as indicated in Table 3. All the berries of each plant were manually picked and pooled together for the same cultivar. Cultivars gave berries of different sizes, ranging from 31 to 99 g/100 berries, with 38 g/100 berries for Noir de Bourgogne. Berries were stored frozen (−40 °C) until analysis. The duration of storage ranged from one to three months (Table 3).

### 3.3. Analysis of the Volatile Fraction of Berries

#### 3.3.1. HS-SPME-GC-MS

Volatiles were extracted with 1 cm SPME DVB/CAR/PDMS (divi-nylbenzene/carboxen/polydimethylsiloxane; 50/30 μm) fiber. The fiber (24 Ga 50/30 µm, for manual holder, 3 pK, 57328-U) was purchased from Sigma (Saint-Quentin-Fallavier, France) and used with a manual fiber holder. SPME vials (20 mL, VA201) and septum caps (18-mm caps, 8-mm PTFE/silicon septum, SACA001) were purchased from JASCO (Lisses, France). Exactly 2 g of frozen berries were placed in the vial. They were left to defrost for 30 min at ambient temperature. Through preliminary experiments, the equilibrium step was set to 30 min at ambient temperature and the extraction step when the fiber was exposed to the headspace to 30 min at ambient temperature.

An HP 6890 Series Gas Chromatograph (Waldbronn, Germany) equipped with an HP 5973 Mass Selective Detector (USA) (Quadrupole) was used with a DB-WAX column (30 m × 0.32 mm × 0.25 µm, 123-7032, Agilent, J&W Scientific, Santa Clara, CA, USA) to analyze the compounds of interest. The SPME fiber was de-sorbed and maintained in the injection port at 250 °C for 5 min. The sample was injected with a purge flow of 20 mL/min for 2 min. Helium was used as carrier gas at 1.4 mL/min with a linear velocity of 43 cm/s. The temperature program was isothermal at 40 °C for 10 min, an increase to 100 °C at a rate of 2 °C/min, isothermal at 100 °C for 6 min, an increase to 120 °C at a rate of 4 °C/min, isothermal at 120 °C for 2 min, and then an increase to 240 °C at a rate of 20 °C/min, which was maintained for 5 min. The total run time was 64 min. The ionization source and transfer line temperatures were set at 230 °C and 160 °C, respectively. The mass spectra were obtained using a mass-selective detector with an electron impact voltage of 70 eV in full scan mode over the range from 29 to 400 *m*/*z*.

Analyses were completed in triplicate.

#### 3.3.2. Identification of Compounds

To conduct the data analysis, compounds with areas above 100,000 (TIC intensity corresponding to the peak integration threshold) and known to be odorant were kept.

Identification was based on the analysis of pure compounds: 3-carene, γ-terpinene, ocimene, 1S-α-pinene, β-pinene, and caryophyllene. The other volatile compounds were compared using their mass spectra from several libraries (NIST 08 (National Institute of Standards and Technology), WILEY138, and INRAMASS (personal library)). The identification of these compounds was also verified with retention index calculations.

#### 3.3.3. Selection of Representative Volatile Compounds

Compounds identified for the three replicates of the 2020 harvest were pooled. For the final data analysis, we selected the volatile compounds found at least twice in the three replicates of each cultivar.

Compounds were reported in order of decreasing peak area and decreasing frequency of appearance (which was the percentage for the presence of peaks in the chromatogram). Comparisons between the different cultivars were based on the 34 compounds with the most important peak areas and highest frequencies of appearance (Table 1).

### 3.4. Strategy for Multivariate Data Analyses

The relative standard deviation (RSD) was used to determine whether the “regular” standard deviation was small or large when compared to the mean for a considered dataset. If percentages were large—for instance, more than 55%—this indicated that the data were more spread out. This made it possible to appreciate how precise the data were in the experiment. The more precise the data, the smaller the RSD was.

Univariate statistical analysis and data visualization were used to produce visualizations of information in order to highlight complex data relationships.

Finally, multivariate data analyses were used to explore chemical profiles in more depth and to determine the existence of specific groups. The goal of these analyses was to extract the important information from the data and to express this information as a set of summary indices called principal components. Then, multidimensional scaling (MDS) was used. This dimension-reduction technique was designed to project high-dimensional data down to two dimensions while preserving relative distances between observations. For both MDS and PCA, proximity measures such as the correlation coefficient or Euclidean distance were used to generate a spatial configuration (map) of points in multidimensional space, where the distances between points reflected the similarity of isolates. PCA minimized dimensions, preserving the covariance of data, and MDS minimized dimensions, preserving the distance between data points. MDS was most useful when the observations were significant and relatively small, such as different cultivars [19,20].

Data analyses were carried out using RStudio-1.4.1106 and R-4.0.5 [21,22] and specific packages: FactoMineR [23], Factoextra [24], magrittr [25], dplyr [26], ggpubr [27], corrplot [28], UpSetR [29], reshape2 [30]. FactoMineR, factoextra, and corrplot were used for PCA; UpSetR and reshape2 for creating UpSet plots; and magrittr, dplyr, and ggpubr for MDS.

## 4. Conclusions

Among the 15 cultivars that were cultivated in the same conditions and harvested in the same year, some cultivars showed VOC profiles for their berries that were different from the others:

- The Rosenthal cultivar exhibited a quite flat profile with 21 molecules and was characterized by sabinene, β-phellandrene, and ocimene;
- The cultivars Lositkia, Ben Tiran, and Barchatnaia shared the same characteristics, including having the greatest number of detected molecules;
- The Noir de Bourgogne cultivar did not show specific molecules, but it generally had the highest amounts of several molecules, such as 3-carene, limonene, β-phellandrene, ocimene, NI2, α-terpinolene, and bicyclogermacrene.

No other cultivar in this study seemed able to replace Noir de Bourgogne for the production of liquors. This study should be completed by sensory analyses.

Additionally, it is necessary to study other cultivars or to implement hybridization with Noir de Bourgogne as one of the parents.

## Figures and Tables

**Table 1:** List of volatile compounds detected and identified in the headspace of berries for the 15 different cultivars.

Retention Index	CAS Number	Molecules	Reported in the Literature	Codes	Occ. Freq.	Mean
925	64-17-5	Ethanol	[8]	Eth	87	2.98 × 10^5^
975	643-42-7	Methyl butanoate	[9]	Mbut	93	8.53 × 10^5^
1025	80-56-8	α-Pinene	[10]	aPin	100	2.62 × 10^6^
1030	105-54-4	Ethyl butanoate	[4]	Ebut	100	1.45 × 10^6^
1068	79-92-5	Camphene	[11]	Camp	33	9.23 × 10^4^
1102	127-91-3	β-Pinene	[6]	bPin	98	5.86 × 10^5^
1119	3387-41-5	Sabinene	[12]	Sab	96	6.38 × 10^6^
1140	13466-78-9	3-Carene	[13]	3Car	100	2.77 × 10^7^
1154	99-83-2	α-Phellandrene	[11]	aPhe	89	4.79 × 10^5^
1162	99-84-3	NI1		NI1	11	1.33 × 10^5^
1196	138-86-3	Limonene	[4]	Lim	100	4.79 × 10^6^
1203	555-10-2	β-Phellandrene	[9]	bPhe	100	1.06 × 10^7^
1216	6728-26-3	*Trans*-2-hexenal	[12]	t2hx	82	4.01 × 10^5^
1256	29714-87-2	Ocimene	[10]	Ocim	100	1.91 × 10^7^
1273	586-62-9	α-Terpinolene	[10]	aTerno	100	2.41 × 10^7^
1371	111-27-3	Hexanol	[14]	Hexo	100	6.15 × 10^5^
1513	489-40-7	4-Aromadendrene		4Aromad	98	4.90 × 10^5^
1558	138-87-4	*Cis*-β-terpineol		cbTerno	78	1.54 × 10^5^
1568	78-70-6	Linalool	[4]	Lin	82	3.65 × 10^5^
1574	76-49-3	Bornyl acetate	[15]	BorA	100	5.25 × 10^5^
1580	87-44-5	Caryophyllene	[9]	Cary	100	1.87 × 10^7^
1587	475-20-7	NI2		NI2	93	8.95 × 10^5^
1622	489-39-4	Aromadendrene		Aro	100	1.25 × 10^6^
1627	339154-91-5	γ-Elemene	[16]	gEle	60	4.91 × 10^5^
1649	6753-98-6	α-Caryophyllene	[13]	aCary	100	4.49 × 10^6^
1671	10208-80-7	Muurolene	[9]	Muu	93	1.06 × 10^6^
1686	23986-74-5	Germacrene D		GermD	87	1.80 × 10^6^
1717	24703-35-3	Bicyclogermacrene	[16]	Bicy	89	1.78 × 10^6^
1760	119-36-8	Methyl salicylate	[13]	Msal	73	4.43 × 10^5^
1789	106-22-9	Citronellol	[9]	Citro	89	8.19 × 10^5^
1825	483-77-2	NI3		NI3	44	4.53 × 10^5^
1848	1197-01-9	*p*-Cymen-8-ol	[13]	pCym8	73	1.68 × 10^5^
1954	23262-34-2	Dendrolasin		Dend	53	1.42 × 10^5^
2127	6750-60-3	Spathulenol	[12]	Spat	84	2.18 × 10^5^

Codes: abbreviation of each molecule used for statistical analysis; Occ. Freq.: appearance frequency of each molecule for the three replicates of each cultivar; Mean: global mean of chromatographic areas, calculated for all cultivars and all replicates, with missing data replaced by the normalized chromatograph detection threshold.

**Table 2:** Stability of peak areas, expressed by the relative standard deviation (RSD).

RSD “Within” for Each Cultivar	NBSAY20	BDSAY20	ANDSAY20	BURSAY20	RNSAY20	LEASAY20	LOSSAY20	OJSAY20	V88SAY20	PCSAY20	BTISAY20	BARSAY20	ROSSAY20	TIBSAY20	TIFSAY20	Mean of the RSD “Within”	RSD “Between”
Volatile Compounds																	
Eth	37	21	54	72	39	30	66	66	53	18		24	37	55		44	92
Mbut	59	75	57	15	37	61	42	86	43	23		49	47	86	60	53	70
aPin	16	7	4	24	22	61	9	41	61	20	21	19	13	46	64	29	73
Ebut	14	52	52	35	31	96	30	76	47	13	15	31	40	71	107	47	88
Camp					21		13					29	10			18	69
bPin	20	46	4	61	27	67	5	58	23	24	43	62	8	76	61	39	89
Sab	16	17	21		59	70	15	53	52	34	24	43	12	68	74	40	150
3Car	13	14	6	19	17	65	9	41	17	25	15	35	88	54	56	32	75
aPhe	20	7	9	10	27	67	7	23		27	6	21		39	40	23	49
NI1																	
Lim	8	6	17	11	4	66	9	42	12	31	10	31	13	78	38	25	84
bPhe	10	12	8	19	11	50	10	41	18	31	14	36	11	59	52	25	103
t2hx		18	37	31	36	87	27	38	24	62	35	17	23		26	35	86
Ocim	54	7	14	12	17	59	7	43	14	32	35	40	20	49	48	30	101
aTerno	9	10	13	14	15	61	13	39	16	32	11	31	15	58	44	25	73
Hexo	11	60	5	15	28	24	33	33	23	99	30	7	12	33	90	34	52
4Aromad	35	53	11	25	52	25	2	41	37	10	9	25	17	43	36	28	84
cbTerno	36	9			17		43	70	58	1	46	13	15	33		31	64
Lin	7	17		23	18	24	18		23	35	11	25		30	14	20	152
BorA	11	35	25	9	37	61	15	78	37	33	44	13	17	28	33	32	152
Cary	13	10	9	8	22	62	13	95	18	47	13	20	25	51	42	30	87
NI2	9	8	13	11	25	63	16	29	45		14	29	22	39		25	161
Aro	53	32	23	10	4	62	43	66	23	63	18	12	11	50	8	32	84
gEle		12			19	50		47	11	38		17		47	38	31	70
aCary	11	9	10	5	18	58	12	40	19	33	10	18	24	44	45	24	126
Muu	16	30	6	50	19	75	22	15	64	9	8	17		89	47	33	114
GermD	9	14	21	10	23	66	19	59			17	35	10	13	52	27	135
Bicy	8	15	11	10	28	65	13	48	30		45	40	28		14	27	158
Msal	20				47		40	7	12	51	10	14	24	37	38	27	139
Citro	9	28	77	2	24	73	13		16	69	7	25		44	26	32	114
NI3	16	12			22				17	43					46	26	66
pCym8	21	48		30	24	46	46	57			24	6		23		33	63
Dend	11			10	3	31		55			7	25			41	23	91
Spat	12	4		9	8	53	13	53	2	25	20	33	20	16		21	90

Molecules with means for the RSD “within” and the RSD “between” colored in black were suppressed for the final multivariate data analysis. Cells colored in gray correspond to molecules with abundances below the detection threshold or molecules found only once among the three replicates.

**Table 3:** List of the cultivars and parameters of cultivation. Size of berries: “heterogeneous” means that berries were of different sizes and “homogeneous” means that berries were globally the same size; maturity of berries: “heterogeneous” means that the maturity and color of berries varied a little and “homogeneous” means all berries were deep black.

Cultivars	Labeling	Earliness	Harvest Date	Analysis Date	Size of Berries	Weight Mean of 1 Berry (g)	Maturity of Berries	Brix Degree at Harvest
Noir de Bourgogne	NBSAY20	Late	24 June 2020	22 July 2020	Homogeneous small	0.38	homogeneous ripe	15.3
Black Down	BDSAY20	Early	29 June 2020	21 July 2020	Homogeneous small	0.64	homogeneous ripe	17.1
Andega	ANSAY20	Middle season	23 June 2020	29 July 2020	Heterogeneous	0.70	homogeneous ripe	18.6
Burga	BUSAY20	Early	18 June 2020	20 August 2020	Homogeneous medium	0.54	homogeneous ripe	14.7
Royal de Naples	RNSAY20	Early	23 June 2020	23 July 2020	Homogeneous small	0.46	homogeneous ripe	19.6
Leandra	LEASAY20	Very late	30 June 2020	20 July 2020	Homogeneous large	0.99	homogeneous ripe	17.6
Lositkia	LOSSAY20	Middle season	25 June 2020	31 July 2020	Heterogeneous	0.50	heterogeneous	19.4
OJ-5-3	OJSAY20	Late	30 June 2020	17 July 2020	Homogeneous large	0.70	homogeneous ripe	16.3
88-04-181	V88SAY20	Early	25 June 2020	30 July 2020	Heterogeneous	0.58	heterogeneous	16.3
PC110	PCSAY20	Middle season	22 June 2020	24 July 2020	Homogeneous large	0.96	homogeneous ripe	18.9
Ben Tiran	BTISAY20	Very late	16 July 2020	23 July 2020	Homogeneous large	0.76	homogeneous ripe	16.8
Barchatnaia	BARSAY20	Early	24 June 2020	27 July 2020	Homogeneous small	0.50	homogeneous ripe	20
Rosenthal	ROSSAY20	Late	25 June 2020	28 July 2020	Homogeneous	0.31	homogeneous ripe	16.4
Tiben	TIBSAY20	Middle season	29 June 2020	28 July 2020	Homogeneous large	0.78	homogeneous ripe	16.3
Tifon	TIFSAY20	Middle season	22 June 2020	21 August 2020	Homogeneous large	0.80	homogeneous ripe	17.4

## Sample Availability

All studied samples are available from the authors.
